# Markers of leucine‐rich repeat kinase 2 activity are associated with pathological protein aggregation across neurodegenerative diseases

**DOI:** 10.1002/alz70855_107317

**Published:** 2025-12-25

**Authors:** Silas A Buck, John F Ervin, Shih‐Hsiu Jerry Wang, Todd J Cohen, Laurie H Sanders

**Affiliations:** ^1^ Duke University Medical Center, Durham, NC, USA; ^2^ University of North Carolina, Chapel Hill, NC, USA

## Abstract

**Background:**

Alzheimer's disease (AD) and primary tauopathies are characterized by pathological tau aggregation, in the form of neurofibrillary tangles and dystrophic neurites. Recent evidence suggests tau pathology is also commonly observed in synucleinopathies as well, including Parkinson's disease (PD) and Dementia with Lewy Bodies (DLB). Interestingly, mutations in the leucine‐rich repeat kinase 2 (*LRRK2*) gene cause autosomal dominant PD, and both alpha‐synuclein and tau pathology are commonly observed in LRRK2 PD. To date, however, direct evidence linking LRRK2 to alpha‐synuclein or tau pathology in human neurodegenerative diseases has not been described.

**Method:**

Since PD‐causing LRRK2 variants display increased kinase activity, we investigated whether levels of LRRK2 kinase activity markers correlate with pathology by performing immunohistochemical labeling in brains from subjects with AD, DLB, idiopathic PD (iPD), LRRK2 PD, and unaffected age‐matched controls. We used phospho‐Rab10 T73 (pRab10) and phospho‐Rab12 S106 (pRab12) as markers of LRRK2 kinase activity. For follow‐up studies, immunofluorescence of pRab12 was performed in PS19 mice, which overexpress human pathological mutant tau in neurons.

**Result:**

We found that pRab12‐positive area increases in AD, DLB, iPD, and LRRK2 PD, localizing to tau aggregates in AD and both tau aggregates and Lewy bodies in DLB, iPD and LRRK2 PD. In addition, we observed labeling of granulovacuolar degeneration bodies (GVBs) in AD, DLB, iPD and LRRK2 PD by both pRab10 and pRab12. We replicated this pRab12 GVB labeling in PS19 mice, which display age‐related accumulation of hyperphosphorylated tau, demonstrating that pathological tau triggers GVBs that are pRab12‐positive. Further, we found that lysosomal marker LAMP1 labels an outer membrane surrounding this pRab12‐positive dense core, confirming previous reports that GVBs are lysosomal structures. Finally, these pRab12‐positive GVBs co‐localized with phosphorylated tau (AT8) and mitophagy marker phospho‐ubiquitin S65, implicating tau processing and mitophagy in pRab12‐associated GVB formation.

**Conclusion:**

In conclusion, phosphorylation of Rab proteins by LRRK2 may play a role in pathological tau and GVB accumulation across neurodegenerative diseases.

